# Supplementary material for: A high precision laser scanning system for measuring shape and volume of transtibial amputee residual limbs: Design and validation
Source: PLoS One. 2024 Jul 11;19(7):e0301619. doi: 10.1371/journal.pone.0301619 (PMC11239001; doi:10.1371/journal.pone.0301619)
Supplement: Attachment — Submitted filename: Response to Reviewers v3.docx [file pone.0301619.s001.docx]

*>>> The authors would like to thank the reviewers for their time in reviewing the article and their valuable suggestions. We have made every attempt to fully address the concerns noted in the reviews. Below you will find our point-by-point responses to each Editor and reviewer comment. We have indicated our responses by “>>>” and italics. We have also highlighted in yellow any changes to the paper itself as a result of addressing these comments.*

General Editor Comments

Please remove any funding-related text from the manuscript and let us know how you would like to update your Funding Statement.

*>>> It has been removed from the manuscript.*

Let us know how you would like to update your Funding Statement.

*>>> “This material is based upon work supported by the National Science Foundation under Grant No. 1906132.   The funders had no role in study design, data collection and analysis, decision to publish, or preparation of the manuscript.”*

We note that you have stated that you will provide repository information for your data at acceptance. Should your manuscript be accepted for publication, we will hold it until you provide the relevant accession numbers or DOIs necessary to access your data.

*>>> Thank you.*

Please include your full ethics statement in the ‘Methods’ section of your manuscript file. In your statement, please include the full name of the IRB or ethics committee who approved or waived your study, as well as whether or not you obtained informed written or verbal consent. If consent was waived for your study, please include this information in your statement as well.

*>>> We have added the requested information.*

Additional Editor Comments

A separated file must be provided for the authors’ answers to the comments, one by one. Moreover, all changes must be yellow-colored highlighted sentences in the revised article. The track changes condition is not suggested.

*>>> We have included our responses to reviewer comments here, and highlighted additions to the article in yellow.*

Using references suggested by the reviewers is not mandatory. If they are related, they could be used by the authors. If not, they could be ignored by the authors.

All keywords must be found in the abstract or the title.

*>>> We have confirmed that all keywords confirmed can be found in the abstract or title.*

The novelty of the manuscript must be highlighted in the introduction, compared to the literature review.

*>>> Extra content in the introduction was added that compare the system in this work with previous works.*

All formulations need references, unless they were extracted or introduced by the authors.

*>>> All formulations were derived by the authors and no references were used.*

The background color of Figures 15, 19, and 20 must be white. The scale bar must be also added.

*>>> We have changed the background color of these figures to white. We are unsure how to create a color scale bar for the images as the colors (e.g. red, orange) are only used to distinguish between two different scans and have no numerical value.*

The digit numbers must be similar in Tables 2 and 4.

*>>> The Table were formatted to be consistent with all other tables. The number of decimal places is consistent throughout the table.*

The conclusion section should be rewritten one by one, in bullets, to show the novelty.

*>>> The conclusion has been expanded using a bulleted point list.*

The discussion is poor and it must be improved. They must be compared to other results of other similar articles.

*>>> We have added measures of reliability of scan volume to our Results, and added to the Discussion a comparison of these results to other studies.*

References should be updated based on recent articles, published in 2015-2023. Moreover, it should be extended to at least 35 articles for a proper discussion.

*>>> We added the most recent articles on the subject of scanning the residual limb, including a short comment on each article.*

*Bailey-Brændgaard, M., and Enevoldsen, P. W. "Accuracy and Reliability of 3D Scanning Spatial Data when Capturing Limb Morphology for Use within Prosthetics and Orthotics: A Scoping Review," 2022.*

*Dickinson, A. S., Donovan-Hall, M. K., Kheng, S., Bou, K., Tech, A., Steer, J. W., Metcalf, C. D., and Worsley, P. R. "Selecting appropriate 3D scanning technologies for prosthetic socket design and transtibial residual limb shape characterization," JPO: Journal of Prosthetics and Orthotics Vol. 34, No. 1, 2022, pp. 33-43.*

*Kofman, R., Winter, R. E., Emmelot, C. H., Geertzen, J. H., and Dijkstra, P. U. "Clinical usability, reliability, and repeatability of noncontact scanners in measuring residual limb volume in persons with transtibial amputation," Prosthetics and Orthotics International Vol. 46, No. 2, 2022, pp. 164-169.*

*Ngan, C. C., Sivasambu, H., Ramdial, S., and Andrysek, J. "Evaluating the Reliability of a Shape Capturing Process for Transradial Residual Limb Using a Non-Contact Scanner," Sensors Vol. 22, No. 18, 2022, p. 6863.*

*Rodrigues, A. S., Oliveira, M. C., and Da Gama, A. E. "Anthropometric Evaluation of Two Low-Cost 3D Digitizers for Lower Limb Prosthetics: A Preliminary Study," 2022.*

*Seminati, E., Young, M., Canepa Talamas, D., Twiste, M., Dhokia, V., and Bilzon, J. "Reliability of three different methods for assessing amputee residuum shape and volume: 3D scanners vs. circumferential measurements," Prosthetics and Orthotics International Vol. 46, No. 4, 2022, pp. 327-334.*

Reviewer #1:

The paper focuses on developing and validating a 3D laser scanner for measuring the volume and shape of residual limbs among amputees.

The introduction needs to discuss previous studies on using laser scanning for measuring residual limb shape and volume, along with their limitations.

*>>> The introduction has been expanded with a discussion on other scanning systems with their limitations.*

Make the figure captions more concise while ensuring clarity, for example in Figure 5. Please also check for typos.

*>>> Edits were made to the captions to make them more concise.*

Explain the parameters used in Figures within the main text (for example, in Figure 7). Clarify the differences between the flat calibration object and the flat plate.

*>>> We feel the current explanation of the parameters in the Figure 7 caption is reasonable and prefer to keep them there. We have also added explanations of parameters in the Figure 1 caption.*

Explain the reasoning behind choosing a 2Hz excitation frequency for evaluating the motion correction system. Also, clarify why excitation was done in the y-direction.

*>>> Sentences were added to the text to explain these two questions.*

It is suggested to explore alternative methods to verify result accuracy beyond inter-data variability analysis.

*>>> We have added measurements of typical error of the measurement (TEM), intraclass correlation coefficient, and reliability coefficients—all of which have been used by other papers to quantify the performance of various scanner systems. We have also added a comparison of these measurements from our scanner with other scanners reported in the literature.*

Reviewer #2:

This paper presents a nice novel method for automated 3D scanning of a residual limb towards prosthetic socket design, which claims improvements over handheld scanners and shows some novelty over prior rotating/armature-mounted scanners (i.e. not just rotating around limb axis but also covering tip, and spiral path). The authors should be complemented on a very detailed disclosure of method enabling reproducibility.

My main concern is around the many gaps in the literature review meaning that the scientific background is not adequately acknowledged and the clinical need is unclear.

The most recent cited paper was published in 2019 and there has been a lot of work since, in validating hand-held scanners. While hand-held scanners will likely never be as controlled and user-independent as an automated device like the one presented, we do need to consider what is ‘good enough’ and what is practical, low cost, reliable and unintimidating for patients in clinics.

The claimed shortcomings of scanning are based on the paper’s reference [17], around user variability; is this really still the case though? [17] is from 2010 and newest reference is from 2019, which misses much more recent work e.g. DOI:10.1109/TBME.2019.2895283 from 2019 and DOI: 10.1097/PXR.0000000000000105, <https://doi.org/10.3390/s22186863> and DOI: 10.1097/JPO.0000000000000350 all from 2022 and tested with living participants, and the latter providing a comparison with hands-on casting for clinically-successful context. These scanners are now showing clinically valid results which are near or better than limit of detectible volume change and better than plaster casting. There are also several missing studies from earlier in the development of CAD/CAM (see e.g. McGarry and colleagues from Strathclyde – not this reviewer ) who did a lot of foundational work on assessment vs. cylindrical phantoms in the 2000s.

*>>> Thank you for these suggested papers. We have updated our Introduction by including more recent work suggested by the reviewer.*

Also, a more fundamental question - if showing a new system and method, why not compare its capabilities to a clinically-standard hand-held scanner?

*>>> We agree that this would have been an intriguing addition to the paper. However, the resources to obtain a clinically-standard hand-held scanner were not available. In lieu of this, and based on comments from reviewers 1 and 3, we have added to our Discussion a comparison between the current scanner and others described in the literature.*

The study is also very light on acknowledged limitations and requirements for future work, especially with a view to clinical application. For example, the present study covers calibration and validation with residual limb analogue objects nicely, and shows a real-life case study, but the above mentioned papers often considered multiple test participants, enabling characterization of the effect of e.g. measurement size upon error or variability. Factors like packaging and development engineering to take this from a research prototype to a clinically usable device, should be noted.

*>>> We have added measures of reliability of scan volume to our Results, and added to the Discussion a comparison of these results to other studies. We have also added to our discussion of limitations and future work.*

Reviewer #3:

Hi,
Evidence for the innovation of the article has not been provided, so it is suggested that a comparison be made with similar researches and the advantages of this method be expressed with them. For example, compare the amount of errors with reference (25. Seminati E, Canepa Talamas D, Young M, Twiste M, Dhokia V, Bilzon JL. Validity and
reliability of a novel 3D scanner for assessment of the shape and volume of amputees’ residual
limb models. PloS one. 2017;12(9):e0184498.).

Statistical analysis is incomplete and the data should be provided to the reader in the form of tables or graphs.

*>>> We have added measures of reliability of scan volume to our Results, and added to the Discussion a comparison of these results to other studies. Thank you for the suggested reference. We have added this to our manuscript and compared our results to it.*
